# Case Report: Autologous stem cell transplantation in a patient diagnosed with AL amyloidosis in Kazakhstan

**DOI:** 10.3389/fonc.2026.1685050

**Published:** 2026-04-22

**Authors:** Olga Kolesnikova, Vadim Kemaykin, Zhandos Burkitbayev, Azat Karabekov, Alexandr Kolesnev, Ayagul Ainabay, Vildanova Ruzal, Meiramgul Yusupova, Fariza Shokubaeva, Akbota Tursyn, Zhuldyz Kuanysh

**Affiliations:** 1 Center for Oncohematology and Bone Marrow Transplantation, National Research Oncology Center, Astana, Kazakhstan; 2 Chairman of the Board, National Research Oncology Center, Astana, Kazakhstan; 3 Department of Scientific Management, National Research Oncology Center, Astana, Kazakhstan

**Keywords:** AL amyloidosis, autologous hematopoietic stem cell transplantation (auto-HSCT), case report, daratumumab, free light chain, high-dose melphalan

## Abstract

**Introduction:**

AL amyloidosis is a rare plasma cell disorder that causes progressive organ dysfunction due to extracellular deposition of misfolded immunoglobulin light chains. Reports from Central Asia are scarce, and there is limited documentation of successful integration of daratumumab-based therapy with autologous hematopoietic stem cell transplantation (auto-HSCT) in this region.

**Case presentation:**

A 65-year-old woman presented with progressive left supraclavicular lymphadenopathy. Examination revealed bilateral cervical and supraclavicular lymphadenopathy with an ECOG performance status of 1. Laboratory testing showed elevated κ free light chains (50.96 mg/L; κ/λ ratio 12.0). Lymph node biopsy with Congo red staining revealed amyloid deposits with apple-green birefringence, and immunohistochemical protein typing confirmed AL amyloidosis type kappa.

**Interventions and outcomes:**

The patient received four cycles of bortezomib–cyclophosphamide–dexamethasone without hematologic response, followed by two cycles of daratumumab–cyclophosphamide–bortezomib–dexamethasone, achieving a very good partial response (dFLC reduced from 46.7 to 11.9 mg/L) and renal recovery (GFR from 21 to 71 mL/min). She underwent high-dose melphalan conditioning and auto-HSCT, with neutrophil engraftment on day +19 and grade 3 mucositis as the main complication. At one-year follow-up, she remains in hematologic and organ remission on monthly daratumumab maintenance.

**Conclusion:**

This case was selected for reporting due to several distinctive clinical and organizational aspects. AL amyloidosis remains underdiagnosed in Central Asia, and experience with daratumumab-based regimens combined with autologous hematopoietic stem cell transplantation (auto-HSCT) is limited in this region. At our center, patients with suspected plasma cell dyscrasias frequently present with advanced or atypical disease manifestations, often leading to diagnostic delays. Compared with other AL amyloidosis cases treated at our institution, this patient demonstrated an uncommon presentation dominated by extensive extramedullary lymph node involvement, absence of overt cardiac manifestations, and prolonged diagnostic latency. Furthermore, the case illustrates the feasibility of integrating modern induction therapy with daratumumab, successful stem cell mobilization, and auto-HSCT in a resource-variable healthcare setting.

## 1 Introduction

Immunoglobulin light chain (AL) amyloidosis is characterized by a clonal population of immunoglobulin secreting cells that produces a monoclonal light chain of κ or λ type as either an intact molecule or a fragment. Amyloidosis can be associated with any B-cell neoplasm that secretes immunoglobulins, including CLL, macroglobulinemia, and non-lymphoplasmacytic lymphoma (1). The light chain protein, instead of conforming to the α-helical configuration of most proteins, misfolds and forms a β-pleated sheet. An increase in serum levels of free light chains precedes the development of AL amyloidosis by many years (2). This insoluble protein deposits in tissues and interferes with organ function. Circulating soluble light chains may also be directly toxic to tissues such as the heart. The β-pleated sheet configuration is responsible for positive staining with Congo red when viewed under polarized light; this staining is required for the diagnosis of AL amyloidosis. It is a relatively rare condition, with a worldwide incidence of 5.1 to 12.8 cases per million person-years (3). In 2018, an estimated 74 000 AL amyloidosis cases worldwide were diagnosed over 20 years. The incidence and 20-year prevalence rates were 10 and 51 patients per million population, respectively. AL is responsible for 0.8% of end-stage renal disease. The median age at diagnosis was 76 years, and one would expect 3800 new cases in the United States each year. There appear to be geographic and racial disparities in the diagnosis (4, 5).

Adequate technology and expertise are needed for a prompt and correct diagnosis, particularly for ruling out non-AL amyloidoses that are now also treatable. Therapy should be carefully tailored based on severity of organ involvement and clonal characteristics, and early and continual monitoring of response is critical. (6, 7).

This case is distinctive for several reasons. First, the disease presented predominantly with extensive extramedullary lymph node involvement, an uncommon manifestation of AL amyloidosis that can mimic lymphoproliferative disorders and delay diagnosis. Second, despite prolonged diagnostic latency, the patient had no clinically significant cardiac involvement, allowing the use of intensive therapeutic strategies. Third, the case illustrates the feasibility of sequential daratumumab-based induction, successful stem cell mobilization, and autologous hematopoietic stem cell transplantation in a resource-variable setting, which remains underreported in the literature from Central Asia (8).

## 2 Case description

Patient, a 65-year-old woman, first noticed an enlarged lymph node in the left supraclavicular region in 2020. In 2021, due to progressive enlargement, patient consulted an oncologist; a lymphoproliferative disease was suspected, and she was referred to the Department of Oncohematology and Bone Marrow Transplantation (BMT) of the National Research Oncology Center (NROC) in Astana. At presentation, she reported no active complaints. Physical examination revealed enlarged jugular and supraclavicular lymph nodes bilaterally, and her ECOG performance status was 1. Biochemical analysis demonstrated elevated serum creatinine. There was no known genetic predisposition, family history was unremarkable, and her past medical history was notable for arterial hypertension. Given the presence of progressive lymphadenopathy, the differential diagnosis initially included lymphoproliferative disorders such as non-Hodgkin lymphoma, plasma cell myeloma with extramedullary involvement, and other forms of systemic amyloidosis. A lymph node biopsy was performed, according to the result, the morphological picture characterizes the substrate of plasma cell myeloma kappa+, with massive extramedullary lesion of the lymph node with AL amyloidosis, kappa+. Histochemical staining with Congo-red revealed congophilic masses with apple-green fluorescence under polarized light. Immunohistochemical (IHC) analysis was performed on sections of a lymph node obtained from a paraffin block. The results demonstrated a prominent mature plasma cell component expressing CD138 (membranous staining), IgG (cytoplasmic staining), and cyclin D1 (uniform nuclear expression, indicating CCND1 gene rearrangement). Light chain restriction with kappa predominance was observed (lambda-positive cells were rare). The plasma cells were CD19-negative, based on correlation of immunohistochemical architecture with CD20 and CD138 staining. Small B cells (CD20+, CD19+) were identified in small aggregates interspersed among the mature plasma cell component (CD138+, CD20−, CD19−/+). No IgA-positive cells were detected.

A bone marrow biopsy was also performed. Histological examination revealed a hypocellular bone marrow (relative to age-adjusted norms) with focal areas of increased cellularity. There was a marked interstitial and focal infiltration by mature plasma cells among the myelopoietic elements.

Hematopoiesis was represented by all three lineages, with an overall decrease in cellularity. The granulocytic lineage showed predominance of mature forms. Erythroid precursors were of normoblastic type. Megakaryocytes were of normal and small size, with hypo- and hyperlobulated nuclei.

Scattered small lymphoid cells were present in the interstitium, along with a small reactive aggregate. IHC analysis for immunoglobulin light chains demonstrated kappa light chain restriction in the plasma cell infiltrate (lambda-positive cells were rare).

Congo red staining with evaluation under polarized light showed no evidence of amyloid deposition.

According to the results of the laboratory examination, the complete blood count values are within normal limits, in the biochemical blood test: serum creatinine 1.8 mg/dL, blood urea 36 mg/dL, albumin 3.6 g/dL, alkaline phosphatase 52 U/L, free light chain κ 50.96 mg/L, κ/λ ratio 12.0, and differential free light chain (dFLC) 46.7 mg/L. Immunofixation of serum revealed a monoclonal paraprotein IgG/kappa. The concentration of the paraprotein is 12.9 g/L. Immunofixation of protein in urine revealed a paraprotein represented by the free kappa chain, the concentration of which is 0.21 g/day. In flow cytometry, in the examined bone marrow sample, the population of cells with the immunophenotype: CD138+/CD38+/Kappacyt-/Lambdacyt-/CD56+/CD19-/CD20-/was 5%.

In April 2022, approximately 24 months after the onset of symptoms, Kappa light chain amyloidosis stage I Revised Mayo Clinic System, 2012. Stage II Palladini G, 2014 was diagnosed. Cytogenetic and molecular studies were not performed due to limited local availability at the time of diagnosis.

In this case, high-dose chemotherapy followed by peripheral stem cell transplantation is considered the most effective. In favor of treatment, a decision was made to conduct a standard first-line regimen based on bortezomib, dexamethasone and cyclophosphamide (VCD). After four cycles of chemotherapy, no hematological response was achieved. Taking into account that a complete response was not achieved and the availability of the drug daratumumab in the clinic, it was decided to add this drug to the patient’s treatment regimen in accordance with the recommendations for the treatment of this disease. The dynamics of hematologic and renal laboratory parameters during treatment are summarized in **Table 1**. Despite initial VCD therapy, no meaningful hematologic response was observed, whereas daratumumab-based resulted in a rapid reduction of dFLC and subsequent renal recovery.

**Table 1:** Dynamics of key laboratory parameters during treatment.

Time point	κ FLC (mg/L)	λ FLC (mg/L)	κ/λ ratio	dFLC (mg/L)	Paraprotein IgG κ (g/L)	Creatinine (mg/dL)	GFR (mL/min)
At diagnosis	50.96	4.25	12.0	46.7	12.9	1.8	21
After 4 cycles VCD	45.2	4.3	10.5	40.9	3.1	1.6	28
After 2 cycles Dara-CyBorD	13.4	1.5	8.9	11.9	0.3	1.2	71
Pre-auto-HSCT (progression)	17.3	4.1	4.2	13.2	8.9	1.5	39
Day +90 post-auto-HSCT	9.1	5.2	1.75	3.9	Not detected	0.9	83

A treatment response was achieved, hematopoietic cell (HSC) mobilization was performed using the etoposide + G-CSF regimen (9), and a sufficient number of cells were harvested. Pending cell availability in the transplant department, maintenance therapy with daratumumab monotherapy was initiated. During one cycle, the patient developed pneumonia, probably caused by Pneumocystis carinii, which was successfully treated with antibacterial therapy (sulfamethoxazole/trimethoprim), and maintenance therapy was stopped. A follow-up examination after 6 months revealed disease progression, and two courses of chemotherapy were administered using the Dara-CyBorD regimen. After two cycles, a very good partial response (VGPR) was achieved.

Conditioning with high doses of melphalan 200 mg/m2 was performed. Then, autologous hematopoietic stem cells a total dose of 4.6 × 10^6^ CD34+ cells/kg was infused. Neutrophilic engraftment occurred on day +19. In the post-transplant period, there were complications in the form of grade 3 mucositis.

The patient was discharged on day +25 after bone marrow transplantation under the supervision of a hematologist at the place of residence with recommendations for control restaging on day +90 in the conditions of the transplant center.

In January 2024, she was hospitalized in the Department of Oncohematology and BMT of the NROC to evaluate the effect of Auto-HSCT. The patient demonstrated a significant clinical improvement in the form of normalization of the sizes of all groups of lymph nodes, improvement of renal function (GFR from 21 to 83.2 mL/min, creatinine and urea within reference values), and an important result was normal values and the ratio of free light chains of immunoglobulins in the serum. She noted significant improvement in physical strength and quality of life after therapy, despite challenges during pneumonia treatment and post-transplant recovery. The patient is receiving maintenance therapy with subcutaneous daratumumab 1800 mg once a month. Follow-up examinations show sustained hematologic and organ responses.

## 3 Discussion

Lymph node-dominant extramedullary involvement is a rare presentation of AL amyloidosis and represents a significant diagnostic challenge. In the absence of classic cardiac or renal red flags, such cases may be misclassified as indolent lymphoma or plasma cell neoplasms with nodal involvement. This case underscores the importance of considering AL amyloidosis in the differential diagnosis of unexplained lymphadenopathy and highlights the critical role of Congo red staining in establishing the correct diagnosis.

Al-amyloidosis is associated with clonal plasma cells that produce abnormal immunoglobulins, which subsequently fold abnormally into amyloid fibrils and are deposited in vital organs, causing organ dysfunction. Most patients seek specialist advice and are diagnosed more than a year after the onset of symptoms similar to our patient (10). The prognosis is determined by the involvement of vital organs, especially the heart (11, 12).

The treatment of AL amyloidosis is primarily aimed at eliminating the clonal plasma cells responsible for light chain production. Melphalan with steroids were the first drugs used to treat amyloidosis (13). Given the efficacy of proteasome inhibitors in targeting clonal plasma cells, bortezomib has also been used in amyloidosis. The standard of care included a combination of a proteasome inhibitor (PI) (bortezomib), an alkylator (cyclophosphamide), and a steroid (dexamethasone) (VCD) (14, 15). In the largest retrospective series of 230 patients treated with VCD, the overall hematologic response rate (ORR) was 60% (complete response [CR] 23%). Organ response was optimal but often delayed (16). In 2015, the US Food and Drug Administration (FDA) approved an anti-CD38 monoclonal antibody (daratumumab) for the treatment of relapsed/refractory multiple myeloma (16) (17),. The addition of the monoclonal antibody resulted in a significant improvement in ORR (92% vs 77%; CR 53% vs 18%), and progression-free survival for major organ failure favored the quadruplet arm (hazard ratio for major organ failure, hematologic progression, or death, 0.58; 95% CI, 0.36-0.93; P = .02); the quadruplet arm was also associated with a doubling of responses at 6 months (cardiac response, 42% vs 22%; renal response, 53% vs 24%) (18).

Achieving an early and profound response to therapy in patients with AL amyloidosis is of utmost importance. Palladini et al., showed the importance of an early hematological response at 3 months as a predictor of survival in all patients with AL amyloidosis (19). This was also true in patients with very advanced cardiac disease (stage IIIB disease, NT-proBNP >8500 ng/L). Patients achieving CR/Very Good Partial Response (VGPR) at the end of the first month of therapy demonstrated improved survival in this group. However, the optimal endpoint of therapy remains controversial and new definitions of profound hematological responses have been proposed (20). Among patients treated in the ANDROMEDA study, those receiving dara-VCD were more likely to achieve complete recovery and free light chain (FLC) <20 mg/L or dFLC <10 mg/L. The clinical relevance of minimal residual bone marrow disease (MRD) assessment and its relationship to organ responses is also of great interest but has not been studied in AL amyloidosis. In some patients, treatment intensification with high doses of melphalan is possible. However, in single treatment, survival in one third of patients in several groups exceeded 15 years (21). With a better understanding of disease biology, improved supportive care and refined patient selection criteria, treatment-related mortality is now less than 3% (22). When auto-HSCT is used as a consolidation strategy after induction, this approach is associated with a deeper response and improved overall and progression-free survival (23). Similar experiences underscore both the therapeutic potential and the complex clinical challenges associated with transplantation in plasma cell-derived malignancies (24).

Patients who are not candidates for Auto-НSCT, other plasma cell-directed therapies have shown efficacy. Ixazomib, an oral PI with a manageable safety profile, generated much interest when a phase 1/2 study reported responses of 52% ORR and 56% organ response in patients treated with 4 mg for up to 12 cycles of therapy. These data led to a randomized phase 3 trial comparing ixazomib with dexamethasone in relapsed patients. Although the study did not significantly improve the overall hematologic response rate, which was the primary endpoint, the duration of hematologic response, vital organ progression-free survival, and time to next treatment were superior in the ixazomib group (25).

Immunomodulatory agents are the mainstay of therapy for multiple myeloma, but their inclusion in AL amyloidosis regimens is challenging due to their unfavorable safety profile in this group. The dosage of both lenalidomide and pomalidomide must be significantly reduced to ensure tolerability (not exceeding 15 mg for lenalidomide and 2 mg for pomalidomide in most patients). At these doses, ORRs of 50% to 70% have been achieved in different series (26–28). Unfortunately, renal dysfunction, cardiac arrhythmias, and elevated cardiac biomarkers have constrained the use of immunomodulatory agents in this group.

Although cytogenetic testing was not performed in this patient, recurrent abnormalities such as t(11;14) are frequently observed in AL amyloidosis and may influence treatment response and transplant outcomes, as reported in the literature. In particular, patients harboring t(11;14) have demonstrated inferior responses to bortezomib-based regimens but favorable outcomes with high-dose melphalan followed by autologous stem cell transplantation. Recurrent cytogenetic abnormalities are present in clonal plasma cells in AL amyloidosis, with t(11;14) being the most common abnormality. Compared with an incidence of 15% to 20% in multiple myeloma, it is present in approximately half of all amyloidosis cases (29). The presence of t(11;14) in AL amyloidosis is associated with worse event-free survival and possibly overall survival when treated with bortezomib, but favorable response rates and event-free survival have been demonstrated when patients with t(11;14) are treated with high-dose melphalan and stem cell transplantation (30). Venetoclax is an orally available selective BCL-2 inhibitor that has shown efficacy in patients with multiple myeloma who have t(11;14) (31). In amyloidosis, several groups have investigated the role of venetoclax in this disease. Premkumar et al. (32) reported on 43 patients (31 with t(11;14)) from 14 centers across the United States and Europe. The ORR was 68% (63% ≥ RR) for the overall cohort and 81% (80% ≥ RR) for the t(11;14) cohort. Among evaluable patients, cardiac and renal response rates were 30% and 40%, respectively, and 10 of 12 responders (88%) had t(11;14).

Antiamyloid antibodies. All the chemotherapy regimens discussed represent anti-plasma cell directed targets. No available treatments target the amyloid fibrils and their soluble toxic precursors. There are now three-phase 3 trials underway using antibodies designed to bind to epitopes on the fibril leading to macrophage activation and fibril dissolution. In a phase 1 trial, CAEL-101 was found to be well tolerated in patients with cardiac AL amyloidosis and led to rapid and sustained organ response in previously treated patients (33). There are now trials underway for patients with cardiac amyloidosis. One is for treatment-naive patients where chemotherapy is combined with the antibody or placebo in patients with cardiac stage IIIA amyloidosis (NCT04512235). The second trial is antibody compared with placebo for stage IIIB cardiac amyloidosis (NCT04504825). The second antiamyloid antibody is birtamimab. (34, 35). In an unplanned *post hoc* analysis, the antibody was found to reduce the risk of death by 50% in Mayo stage IV patients. This has now been activated as a phase 3 trial in newly diagnosed cardiac amyloid patients comparing active antibody plus chemotherapy compared with placebo plus chemotherapy (35, 36).

## 4 Conclusions

This case is noteworthy due to the rare combination of AL amyloidosis with extensive extramedullary lymph node involvement, successful induction with Dara-CyBorD, and consolidation with Auto-HSCT, resulting in both hematologic and organ response. The strength lies in the detailed longitudinal follow-up and comprehensive reporting of laboratory, imaging, and histological data. In selected patients with AL amyloidosis, rapid attainment of deep hematologic response through daratumumab-containing regimens, followed by consolidation with Auto-HSCT, can lead to sustained organ recovery and long-term disease control. This case reinforces the importance of early aggressive therapy, careful patient selection, and multidisciplinary management in optimizing outcomes.

## Data Availability

The raw data supporting the conclusions of this article will be made available by the authors, without undue reservation.
